# Normal tension glaucoma: A dynamic optical coherence tomography angiography study

**DOI:** 10.3389/fmed.2022.1037471

**Published:** 2023-01-06

**Authors:** Jan Van Eijgen, Alexander Heintz, Claire van der Pluijm, Margaux Delporte, Dries De Witte, Geert Molenberghs, João Barbosa-Breda, Ingeborg Stalmans

**Affiliations:** ^1^Department of Neurosciences, Research Group of Ophthalmology, KU Leuven, Leuven, Belgium; ^2^Department of Ophthalmology, University Hospitals UZ Leuven, Leuven, Belgium; ^3^Leuven Biostatistics and Statistical Bioinformatics Centre (L-BioStat), KU Leuven, Leuven, Belgium; ^4^Department of Surgery and Physiology, Cardiovascular R&D Centre - UnIC@RISE, University of Porto, Porto, Portugal; ^5^Department of Ophthalmology, Centro Hospitalar Universitário de São João, Porto, Portugal

**Keywords:** glaucoma, retina, microvasculature, optical coherence tomography angiography, sympathomimetic stimulus, hand grip test

## Abstract

**Purpose:**

Vascular dysregulation seems to play a role in the pathogenesis of glaucoma, in particular normal tension glaucoma (NTG). The development of optical coherence tomography angiography (OCTA) enabled the measurement of the retinal microvasculature non-invasively and with high repeatability. Nonetheless, only a few studies transformed OCTA into a dynamic examination employing a sympathomimetic stimulus. The goal of this study was to use this dynamic OCTA exam (1) to differentiate healthy individuals from glaucoma patients and (2) to distinguish glaucoma subcategories, NTG and high-tension primary open angle glaucoma (POAG).

**Methods:**

Retinal vessel density (VD) in NTG patients (*n* = 16), POAG patients (*n* = 12), and healthy controls (*n* = 14) was compared before and during a hand grip test with a hydraulic dynamometer.

**Results:**

At baseline, mean peripapillary VD was lower in POAG and NTG (42.6 and 48.5%) compared to healthy controls (58.1%; *p* < 0.001) and higher in NTG compared to POAG (*p* = 0.024) when corrected for mean arterial pressure (MAP). Peripapillary and macular (superficial and deep) VD differences were found for gender, age, and baseline MAP. No change in VD occurred (pre-/post-stimulus) in any of the groups.

**Conclusion:**

Retinal VD loss in glaucoma patients was confirmed and the necessity to correct for gender, age and especially MAP was established. Although replication in a larger population is necessary, OCTA might not be the most suitable method to dynamically evaluate the retinal microvasculature.

## 1. Introduction

Glaucoma is the leading cause of irreversible blindness globally and is estimated to affect more than 120 million people by 2040. It leads to a decline of the patient’s visual field is caused by the chronic and progressive loss of the macular ganglion cell layer. Primary open angle glaucoma (POAG) represents 74% of cases and is characterized by high intraocular pressures (IOP; > 21 mmHg) without iridocorneal angle closure or other evident causes of IOP increase (1). Normal tension glaucoma (NTG; IOP ≤ 21 mmHg) is considered a subgroup of POAG and is treated similarly by lowering the IOP, even in the presence of normal IOP. This initially challenged the unquestioned association of glaucoma and elevated IOP (2). According to ethnic background the proportion of NTG can greatly differ, constituting up to 52–92% of POAG patients in Asia and 30–39% of POAG patients with European ancestry (1, 2).

The mechanical theory, for many years considered as mainstay etiology, states that the optic nerve head is mechanically damaged by elevated IOP (3, 4). The mechanical deformation of the lamina cribrosa generates axonal damage along the optic nerve (2). A thinner cornea, higher corneal hysteresis, and a higher translaminar pressure gradient over the lamina cribrosa have been reported to increase the sensitivity of the optic nerve to IOP changes (5, 6).

The vascular theorem claims that the optic neuropathy is elicited by repetitive ischaemia and reperfusion (7–9). Cardiovascular comorbidities such as ischemic heart disease, stroke, hypertension, hyperlipidemia, and metabolic syndrome are more prevalent in POAG, predominantly in NTG (7, 10, 11). Furthermore, increased vasoconstriction and decreased retinal blood flow (RBF) in response to sympathomimetic stimuli [e.g., cold pressor test (CPT) and handgrip test] in NTG and the higher prevalence of NTG in patients with migraine, Raynaud’s disease, and Flammer syndrome are aligned with this theory (12–14). The development of optical coherence tomography angiography (OCTA) emphasized the focus on the vascular theory even further (15). Using OCTA, a significant constriction of the retinal vessels has been measured following a handgrip test in healthy individuals (16).

Autoregulation of the RBF, enabling continuous and stable oxygenation, is mostly dependent on the release of vasoactive metabolites, most importantly no (12, 14), and oxidative stress mediators (17, 18). Fluctuation of RBF, low systemic blood pressure (BP) with nocturnal dips and reduced nocturnal RBF have been reported in NTG (8, 19, 20). Low diastolic BP and reduced RBF in response to a handgrip test have been associated with progressive visual field (VF) defects (13, 21, 22). It is hypothesized that repetitive dipping of the BP in absence of effective autoregulation causes recurrent ischaemic damage, which is subsequently responsible for VF damage (8, 14, 23).

In recent years OCTA has been involved in dynamic measurements in combination with physiological stimuli as hypoxia, flicker light and isometric exercise (13, 24–27). Plexus-specific dilatator responses were seen after flicker stimulation, as well as dilation of the superficial capillary plexus after hypoxia, constriction of the deep capillary plexus after hyperoxia and a blunted response after hypoxia or isometric exercise in diabetes type 1 patients (24–27).

To date, only a few studies have studied the vascular reactivity in POAG and NTG separately (28). In another study comparing healthy eyes with POAG and NTG, baseline vessel density (VD) differed between healthy and glaucoma groups; but not between POAG and NTG (29).

This study aims to confirm the ability to distinguish both glaucoma groups from healthy individuals using OCTA and to, more importantly, distinguish NTG from POAG using OCTA and a sympathomimetic stimulus (handgrip test).

## 2. Materials and methods

### 2.1. Study

#### 2.1.1. Participants

Ethical approval was obtained from the UZ/KU Leuven Ethical Research Commission (ethical approval number S62253). Patients with NTG (maximum untreated IOP ≤ 21 mmHg), high pressure POAG (maximum untreated IOP > 21 mmHg) and healthy controls were recruited from the existing cohort of the Leuven eye study (7). The most severely affected eye was selected in glaucoma patients. Exclusion criteria were: (i) diabetes mellitus, (ii) previous ocular trauma, (iii) myopia > −6D, (iv) hyperopia > 4D, or (v) aberrant BP response [drop of the mean arterial pressure (MAP) > 20 mmHg after hand grip test].

#### 2.1.2. Protocol

Patients were instructed to avoid caffeinated drinks on the day of the examination. Baseline BP, routine ophthalmological examination, and baseline OCTA were obtained. Subsequently, maximal grip force was measured in the dominant arm using the Jamar^®^ hydraulic dynamometer. After a 15-min break, OCTA was repeated 3 min into a handgrip test, in which the patient was instructed to hold at least one-third grip strength for 3–5 min. BP was measured every minute on the contralateral arm and the procedure was interrupted when the diastolic BP exceeded 120 mmHg or in the presence of any other adverse event.

The OCTA scan was made using the Angiovue^®^ software (Optovue^®^, Fremont, CA, USA) providing an automatic quantitative measurement of VD in the fovea, perifovea and parafovea using the macular (6 mm × 6 mm) scan divided into a superficial [inner limiting membrane (ILM) to inner plexiform layer (IPL)] and deep layer [IPL to outer plexiform layer (OPL)]. Following the ETDRS grid, the peri- and parafoveal data were further divided into four quadrants: nasal, superior, temporal, and inferior quadrant. The peripapillary vessel density was measured at the (superficial) “radial peripapillary capillary” level of the optic disc following the Garway-Heath map [six regions: temporal 90°, superotemporal (ST) 40°, inferotemporal (IT) 40°, nasal 110°, superonasal (SN) 40°, and inferonasal (IN) 40° regions] where the temporal and nasal part were divided in half [resulting in eight regions, including the new temporal-superior 45° (TS), temporal-inferior 45° (TI), nasal-superior (NS) 55°, and nasal-inferior (NI) regions 55°]. No IOP-lowering medication was discontinued for this study.

### 2.2. Statistical analysis

Statistical analysis was conducted in SAS9.4. The responses were not normally distributed (Shapiro–Wilk test not shown). The Kruskal–Wallis test was applied to compare unpaired continuous responses between groups. The Chi-squared test was performed in order to examine the presence of an association between categorical variables and group membership. Given the central limit theorem, linear mixed models (LMM) with random intercept (allowing subject-specific values at baseline) were considered to evaluate the effect of the hand grip test on retinal parameters while accounting for age, diagnosis, gender, baseline MAP, as well as their time dependent effect as the extent of BP change during the test. Significance was defined as *p* < 0.05.

## 3. Results

Fifty-one eyes of 51 patients were examined. Four participants were forced to stop within 90 s because of a diastolic BP exceeding 120 mmHg, three experienced adverse effects and two exhibited a paradoxal decrease of their MAP after the hand grip test (−30 and −35 mmHg). These data were excluded (*n* = 9).

An overview of cohort characteristics in terms of disease severity, age and gender can be found in Table 1 (no significant differences between groups). Eventually 16 patients with NTG, 12 with POAG and 14 healthy controls participated. Ages ranged from 47 to 75 years. Few participants used antihypertensive medication: only one healthy participant used a beta blocker together with four NTG patients (one ACE inhibitor, one angiotensin II receptor blocker, one calcium channel blocker and one angiotensin II receptor blocker combined with a beta blocker). No antihypertensive medication featured in the POAG subgroup. Supplementary Table 1 summarizes the IOP-lowering medication and surgical antecedents of the glaucoma subgroups. All glaucoma patients but one NTG patient reported chronic use of topical IOP-lowering medication. Eight NTG patients and seven POAG patients had previous IOP-lowering surgery (phaco-emulsification not included). Healthy participants were free of surgical precedents and topical medication.

**TABLE 1:** Descriptive statistics of the analyzed study cohort.

Group	POAG	NTG	Controls	P1	P2	P3
Number of participants	12	16	14			
Median age in years (interval)	60 (47–75)	67 (52–74)	58 (48–73)	0.424	0.045	0.025
Gender female:male	7:5	8:8	9:5	0.786	0.557	0.278
Median MD in decibels (interval)	−2.35 (−20.1–1.9)	−5.6 (−10–2.9)	NA	0.587	NA	NA

P1: Comparison of POAG patients vs. NTG patients (Kruskal–Wallis test for continuous variables. Chi-square test for categorical variables). P2: Comparison of POAG patients vs. controls (Kruskal–Wallis test for continuous variables. Chi-square test for categorical variables). P3: Comparison of NTG patients vs. controls (Kruskal–Wallis test for continuous variables. Chi-square test for categorical variables). POAG, primary open angle glaucoma; NTG, normal tension glaucoma; MD, mean deviation.

### 3.1. Effect of the handgrip test on arterial blood pressure

At baseline, there was no significant difference in systolic, diastolic, or mean arterial BP between the three populations (Table 2; *p* > 0.2). In all three groups, a significant elevation of both systolic as diastolic BP (Table 2) during the handgrip test was noted (median 32 and 25 mmHg; *p* < 0.001).

**TABLE 2:** Baseline and during handgrip median arterial blood pressures (mmHg).

Group	All groups	NTG	POAG	Controls	P1	P2	P3
MAP baseline (interval)	100 (73–129)	99 (73–121)	105 (89–126)	100 (83–129)	0.236	0.624	0.630
MAP handgrip (interval)	130 (97–168)	128 (97–166)	130 (98–151)	130 (107–168)	0.763	0.870	0.983
MAP difference (interval)	29 (2–80)	30 (2–54)	26 (9–38)	26 (9–80)	0.134	0.724	0.381
SYS BP baseline (interval)	141 (109–183)	139 (109–174)	140 (127–183)	139 (120–183)	0.515	0.913	0.553
SYS BP handgrip (interval)	179 (122–232)	176 (122–210)	178 (130–208)	181 (146–216)	1.000	0.496	0.456
SYS BP difference (interval)	32 (−1–96)	32 (4–63)	32 (−1–50)	32 (11–74)	0.457	0.301	0.809
DIA BP baseline (interval)	82 (55–105)	80 (55–98)	86 (68–98)	84 (56–105)	0.236	0.644	0.417
DIA BP handgrip (interval)	108 (65–145)	110 (82–144)	107 (82–127)	100 (65–145)	0.871	0.683	0.843
DIA BP difference (interval)	25 (−9–72)	28 (−9–54)	23 (8–34)	21 (−5–47)	0.109	0.957	0.392

P1: Comparison of POAG patients vs. NTG patients (Kruskal–Wallis test). P2: Comparison of POAG patients vs. controls (Kruskal–Wallis test). P3: Comparison of NTG patients vs. controls (Kruskal–Wallis test). MAP, mean arterial pressure; SYS BP, systolic blood pressure; DIA BP, diastolic blood pressure; POAG, primary open angle glaucoma; NTG, normal tension glaucoma.

### 3.2. Effect of the handgrip test on vessel density

Tables 3–5 summarize the inferred estimates of peripapillary (Table 3), macular superficial (Table 4; ILM to IPL) and macular deep (Table 5, IPL to OPL) VD parameters during the test. Only cells with significant estimates are shown (*p* < 0.05), others were left blank. The mean VD values and standard deviation of all regions can be found in Supplementary Tables 2, 3. The *p*-values of the LMM models can be found in the Supplementary Tables 4–6. The handgrip effect and matching *p*-values were also described for total retinal thickness parameters in the Supplementary Tables 7–10. The intercepts in Tables 3–5 represent the inferred VD for male healthy participants. If significant, the other variables show an additional effect on the intercept VD. By example, adding the value for gender results in the inferred VD for female healthy participants. The variable time represents the overall effect of the handgrip test. The group (diagnosis) effect can be read from the comparative lines indicated in gray in the tables. The combination variables refer to how a certain variable influenced the VD change during the handgrip test. LMM variable changes can only be separately considered bearing in mind that the other variables -theoretically- need to remain constant.

**TABLE 3:** Optic nerve head vessel density optical coherence tomography angiography (OCTA) adjusted linear mixed model estimates (%).

VD region	All	Disc	Peripapil	SUP Hemi	INF Hemi	NS	NI	IN	IT	TI	TS	ST	SN
Intercept	62.4	67.0	64.3	65.9	62.8	47.2	51.4	61.3	64.8	49.5	70.1	60.0	59.0
Gender			4.6	4.4	4.6	5.7							
MAP_0													
Age													
Diagnosis													
POAG (vs. normal)	−12.9		−15.0	−13.6	−15.9	−13.6	−13.1	−17.1	−26.0	−7.8	−9.3	−19.9	−15.7
NTG (vs. normal)	−8.7		−10.0	−8.5	−12.0	−7.7	−7.9	−14.4	−20.7			−14.2	−6.3
NTG (vs. POAG)	4.3		5.0	5.2		5.9	5.1			4.5	6.2		9.4
Time													
Gender*time											−3.1		
MAP_0*time													
MAPdiff*time													
Age*time													
Diagnosis*time													
POAG (vs. normal)													
NTG (vs. normal)													
NTG (vs. POAG)													

Only significant responses (*p* < 0.05) are reported. Blank cells indicate a non-significant result. The intercept represents the inferred VD for male healthy participants. If significant, the other variables show an additional effect on the intercept VD. E.g., adding the value for gender results in the inferred VD for female healthy participants. The variable time represents the overall effect of the handgrip test. The combination variables refer to how a certain variable influenced the VD change during the handgrip test. LMM variable changes can only be separately considered bearing in mind that the other variables -theoretically- need to remain constant. VD, vessel density; MAP_0, mean arterial pressure at baseline; POAG, primary open angle glaucoma; NTG, normal tension glaucoma; MAPdiff, difference of mean arterial pressure during examination; Peripapil, peripapillary mean; SUP, superior; Hemi, hemisphere. INF, inferior; NS, NI, IN, IT, TI, TS, ST, and SN as explained in the section “2 Materials and methods”.

**TABLE 4:** Macular superficial [inner limiting membrane (ILM)-IPL] vascular plexus vessel density (OCTA) adjusted linear mixed model estimates (%).

Vessel density region (ETDRS)	All	All SUP Hemi	All INF Hemi	FAZ area	Fovea	Para fovea	Para SUP Hemi	Para INF Hemi	Para SUP	Para INF	Para NAS	Para TEM	Peri fovea	Peri SUP Hemi	Peri INF Hemi	Peri SUP	Peri INF	Peri NAS	Peri TEM
Intercept	61.4	62.2	60.5	0.4	21.2	56.6	56.8	56.3	54.9	60.0	51.4	59.9	65.5	67.6	63.3	72.7	64.1	66.9	57.8
Gender																			
MAP_0																			
Age		−0.2							−0.3					−0.3		−0.3		−0.3	
Diagnosis																			
POAG (vs. normal)	−10.1	−9.4	−10.9			−7.8	−7.5	−8.1	−7.8	−7.9	−6.3	−9.0	−10.6	−9.8	−11.4	−12.0	−12.0	−8.5	−10.0
NTG (vs. normal)	−10.1	−9.0	−11.4			−8.7	−7.6	−9.8	−8.4	−10.8	−5.8	−9.9	−10.6	−9.1	−12.1	−10.3	−13.0	−8.6	−10.5
NTG (vs. POAG)																			
Time																			
Gender*time						−3.1	−3.2	−3.0		−2.9	−3.6								
MAP_0*time						−0.1	−0.1	−0.2	−0.2	−0.2	−0.1								
MAPdiff*time				−0.001															
Age*time					0.2														
Diagnosis*time																			
POAG (vs. normal)																			
NTG (vs. normal)																			
NTG (vs. POAG)																			

Only significant responses (*p* < 0.05) are reported. Blank cells indicate a non-significant result. The intercept represents the inferred VD for male healthy participants. If significant, the other variables show an additional effect on the intercept VD. E.g., adding the value for gender results in the inferred VD for female healthy participants. The variable time represents the overall effect of the handgrip test. The combination variables refer to how a certain variable influenced the VD change during the handgrip test. LMM variable changes can only be separately considered bearing in mind that the other variables -theoretically- need to remain constant. MAP_0, mean arterial pressure at baseline; POAG, primary open angle glaucoma; NTG, normal tension glaucoma; MAPdiff, difference of mean arterial pressure during examination; SUP, superior; Hemi, hemisphere; INF, inferior; FAZ, foveal avascular zone; Para, parafovea; NAS, nasal; TEM, temporal.

**TABLE 5:** Macular deep [inner plexiform layer (IPL)-outer plexiform layer (OPL)] vascular plexus vessel density (OCTA) adjusted linear mixed model estimates (%).

Vessel density region (ETDRS)	All	All SUP Hemi	All INF Hemi	Fovea	Para fovea	Para SUP Hemi	Para INF Hemi	Para SUP	Para INF	Para NAS	Para TEM	Peri fovea	Peri SUP Hemi	Peri INF Hemi	Peri SUP	Peri INF	Peri NAS	Peri TEM
Intercept	52.5	49.3	55.8	39.8	54.8	54.8	54.9	52.7	59.7	50.2	56.8	55.1	51.8	58.4	51.7	55.9	49.3	63.1
Gender																		
MAP_0										0.2								
Age										−0.2								
Diagnosis																		
POAG (vs. normal)															−5.8			−5.7
NTG (vs. normal)	−5.2	−4.8	−5.5		−3.7	−4.0		−5.0				−5.9	−5.2	−6.6	−5.7	−6.7		−6.3
NTG (vs. POAG)																		
Time				−20.0														
Gender*time																		
MAP_0*time	−0.2	−0.2	−0.2		−0.2	−0.2	−0.2	−0.3	−0.2	−0.2	−0.2	−0.2	−0.2	−0.2	−0.2	−0.2	−0.3	−0.2
MAPdiff*time																		
Age*time	0.5	0.5	0.5	0.4	0.4	0.4	0.5	0.5	0.5		0.4	0.5	0.5	0.5	0.5	0.5	0.6	0.4
Diagnosis*time																		
POAG (vs. normal)																		
NTG (vs. normal)				−4.0					−4.6	−4.3								
NTG (vs. POAG)				−4.5														

Only significant responses (*p* < 0.05) are reported, blank cells indicate a non-significant result. The intercept represents the inferred VD for male healthy participants. If significant, the other variables show an additional effect on the intercept VD. E.g., adding the value for gender results in the inferred VD for female healthy participants. The variable time represents the overall effect of the handgrip test. The combination variables refer to how a certain variable influenced the VD change during the handgrip test. LMM variable changes can only be separately considered bearing in mind that the other variables -theoretically- need to remain constant. MAP_0, mean arterial pressure at baseline; POAG, primary open angle glaucoma; NTG, normal tension glaucoma; MAPdiff, difference of mean arterial pressure during examination; SUP, superior; Hemi, hemisphere; INF, inferior; Para, parafovea; NAS, nasal; TEM, temporal.

In all peripapillary (inferred average difference for POAG of −12.9%, *p* < 0.001 and for NTG of −8.7%, *p* < 0.001) and superficial macular areas (but the fovea) (POAG and NTG −10.1%, *p* < 0.001), the baseline vessel densities in the NTG and POAG groups were significantly lower than in the control group. No significant difference was found in the VD of the fovea (*p* = 0.406), possibly due to its mainly avascular nature. The stimulus itself did, however, result in a strong decrease in the deep foveal VD (−20%, *p* = 0.025) over all groups, with even more decrease in the NTG group (−4.0 and −4.5%, *p* = 0.012 and *p* = 0.008). NTG patients systemically exhibit a significant higher baseline peripapillary VD compared to their POAG counterparts, especially in the superonasal region (+9.4%, *p* = 0.006), without significant mean deviation (MD) differences. All these baseline differences lose significance when not corrected for the baseline MAP (*p* > 0.05, data not shown). In the deep macular plexus baseline differences were only found in the NTG group compared to healthy controls on average (−5.2%, *p* = 0.023), in the superior paramacular (−5%, *p* = 0.036) and in the perifoveal regions (inferred average −5.9%, *p* = 0.021).

Women showed a slightly higher peripapillary VD (+4.6%, *p* = 0.02) and had a more pronounced decrease of VD during handgrip test in the superficial paramacular plexus (ranging from −2.9 to −3.6%, *p* < 0.048), keeping the other covariates fixed.

Controlling for the other variables, the higher the baseline MAP, the more the superficial paramacular plexus and all regions of the deep perifoveal plexus decreased in VD during the handgrip test (ca. −0.2%/mmHg, highest *p* = 0.022).

Lastly the VD of all deep macular regions increased more (ca. +0.5%/year, highest *p* = 0.003) during the handgrip test the older the participants were, if, again, the other variables are kept constant.

## 4. Discussion

Known for its repeatability and discriminative power, OCTA is finding its way into glaucoma clinical care (30). So far, most OCTA research in glaucoma focussed on non-dynamic VD comparison and most time without access to normative datasets. Overall these studies show a lower circumpapillary VD, reduced prelaminar optic disc perfusion, and lower systolic acceleration of the central retinal artery in patients with glaucoma when compared to healthy controls (7, 31–33). Lower blood flow and VD at the optic disc correlates with glaucoma severity in terms of visual field mean deviation, ganglion cell complex thickness and retinal nerve fiber layer (RNFL) thickness (34). VD is also shown to decrease faster in glaucomatous eyes than in their normal counterparts (35). Even within glaucoma subclasses, the reduction in peripapillary VD is apparent in both NTG and POAG separately (36, 37). In this study, we were able to replicate this baseline, non-dynamic VD differences between glaucoma patients (NTG and POAG) and healthy controls. The key findings are summarized in the synopsis text Box 1.

**BOX 1 Synopsis.**

Previously known

- Baseline peripapillary vessel density in healthy individuals is higher compared to glaucoma patients.

- Baseline peripapillary vessel density is higher in NTG than POAG for a similar level of VF damage.

- The handgrip test induces a vasoconstriction response in the retinal vessels.

- Vascular dysregulation measured following sympathomimetic test is more common found in patients with history of cold hands. This study

- Is the first study comparing OCTA responses between POAG, NTG and healthy eyes after induced BP rise.

- Confirms higher baseline peripapillary vessel density measures in healthy individuals compared to glaucoma patients, and in NTG compared to POAG separately.

- Could not find VD changes with OCTA after BP rise in either of the studied groups.

- Proves the importance of statistical correction of OCTA measures for age, gender and especially MAP.

In accordance to Lommatzsch et al. (nasal peripapillary region) (28) and Scripsema et al. (average perfused capillary density) (29) we also report on slightly higher superficial peripapillary VD in patients with NTG patients compared to POAG ones. Lommatzsch et al. only reached significance nasally, but the trend is visible in all regions (28). The race (Caucasian) and severity of the studied glaucoma groups are more or less similar in these study cohorts and the same device was used (Optovue). Secondly, Bojikian et al. reported on a lower optic disc perfusion of the prelaminar tissue in glaucoma compared to healthy controls, but without differences between NTG and POAG specifically, the latter partly in accordance to our lack of differences of within-disc VD between all groups (31). One might argue that the proclivity NTG discs have for focal defects/notching can explain why VD might be slightly higher in all regions when comparing patients with similar visual field severity. Inferotemporally and superotemporally no differences were detected between NTG and high-tension POAG, which supports this claim since rim loss preferentially occurs in these regions for NTG. It has to be noted that the reverse was found at the Fudan University in Shanghai: lower peripapillary VD in NTG than POAG. Racial differences, higher severity levels of glaucoma (−9.11 and −9.76 dB MD for POAG and NTG, respectively where the focal differences between both groups may diminish) and the lack of BP correction might possibly explain these differences (37).

To our knowledge, we are the first to report on gender differences in VD, namely, higher peripapillary VD in women and lower VD after BP rise in the superficial parafoveal plexus.

In addition, we are the first to report on the importance of MAP measurement and correction thereof in the analysis of OCTA. Baseline VD differences between POAG and NTG only become apparent when corrected for baseline MAP. Baseline MAP was also significantly associated with macular VD changes after BP rise, possibly pointing toward an impaired dilator capacity due to endothelial damage. This strongly advocates to analyze and correct for BP levels in future OCTA research. Simultaneous correction for BP and mean ocular perfusion pressure results in collinearity and should be avoided.

In this paper, age is shown to modulate the effect of the BP rise on the VD of the deep plexus. Higher age gives rise to relatively higher post-stimulus VD. Lin et al. showed a decrease in the retinal VD of the deep vascular plexus in an aging population, corrected for confounders such as sex and controlled hypertension (38). A lower starting point (although not significant in this study) might explain why the VD change is less pronounced in the elderly compared to the young. Further research is needed regarding this point.

Interestingly, we noted a strong decrease of deep foveal VD during handgrip in all groups, even more pronounced in NTG. Further research is needed to clarify the discriminative power of the deep foveal vasculature.

In contrast to the static evaluation of VD described above, we propose a dynamic alternative to assess vascular reactivity following a sympathomimetic stimulus. Isometric exercise, as is the hand grip test, results in BP rise and retinal vasoconstriction in the healthy retina according to the protocol of Sousa et al. (16). This vasoconstrictive response was first reported in healthy individuals by Blum et al. using a retinal vessel analyser (39), later corroborated using a compacted laser Doppler flowmeter (13), and lastly confirmed by Sousa et al. using OCTA (16). We, however, were unable to replicate this vasoconstriction in healthy controls except for the deep foveal vessels (all groups).

Possible explanations for this non-significant finding are multiple: (1) We used a linear mixed model to correct for participant characteristics and absolute BP values, thereby properly taking into account between- and within-patient variability, whereas Sousa et al. did not; (2) Lack of power as denoted in the post how power analysis (14 healthy participants instead of 24 in Sousa’s test), but both the expected decrease of retinal thickness and VD in the glaucomatous groups favor external validity of the test; (3) Patients in our study might not always have maintained one third of their maximal measured force, however, the amplitude of our mean MAP increase (30 mmHg) matches that from Sousa et al. (28 mmHg) (16); (4) Our participants were recruited from the Leuven eye study cohort (10). Therefore, only subjects with diabetes mellitus, ocular trauma and high ametropia were excluded, whereas Sousa et al. excluded hypertensive patients, smokers, and patients using vasoactive drugs. The mean MAP values of the study of Sousa et al. going from a 91 to 118 mmHg, are ca 10 mmHg lower than ours. The Bayliss effect is the immediate constrictive, physiological reaction suggested to be the mechanism for retinal vascular constriction following sympathomimetic stimulation using the handgrip test (16, 39). While we correct for the absolute BP values in our model, chronic arterial hypertension might be responsible for this difference since it results in systemic endothelial dysfunction, thus permanently impaired vasodilation, and therefore (retinal) arteriolar narrowing (40, 41). However, exact data regarding the influence of vasoactive (systemic and topical) drugs and long lasting hypertension on retinal vascular reactivity are lacking (32).

Similar to the hand-grip test, the CPT is another sympathomimetic test used to compare autonomic dysregulation in healthy individuals and glaucoma (42, 43). Next to the Bayliss effect in isometric exercise, an additional underlying mechanism for both tests is thought to rely on an elevation in ET-1 (42). Gherghel et al. reported a significant decrease in flow velocity (retinal flowmeter) in POAG patients, without concomitant BP increase. The absent or blunted BP response following CPT might be the result of autonomic vascular dysregulation (43).

Chou et al. on the other hand showed no significant VD change after CPT (OCTA), neither when categorization was based on a history of cold hands. However, the 5-min waiting period between the end of CPT and the beginning of OCTA measurement could explain the absence of significant VD change. In fact, peripheral vascular change following a sympathetic stimulus has been reported to decline after 1 min. For this reason, we chose not to delay the OCTA exam in our current study. Additionally, it has to be noted that Chou et al. did not differentiate between glaucoma subcategories (POAG/NTG). (42) Contrary to the retinal flowmeter, OCTA cannot directly measure flow velocity (44). As flow velocity might represent haemodynamic changes more directly, this could also explain the absence of a significant haemodynamic response in our study and the study of Chou et al.

First the idea OCTA might not be suited for dynamic evaluation after isometric exercise is contradicted by other studies with significant findings (27). Second it can be argued that autoregulatory capacity could theoretically be better evaluated on larger effector vessels. More recently, Streese et al. published standard operating procedures for dynamic vessel analysis with flicker light stimulation using the dynamic vessel analyzer (DVA) (45). The vascular response measured with DVA is diminished in POAG and is proven to improve after surgical glaucoma treatment such as trabeculectomy or transscleral photocyclocoagulation (46, 47). DVA constitutes therefore a promising candidate for future dynamic studies in glaucoma given the multitude of dynamic parameters that can be extracted from its continuous measurement during flicker light stimulation.

### 4.1. Limitations

Due to the relatively small sample size, our study might not have had the power to pick up all VD changes, specifically for the healthy controls. Secondly, IOP was not measured during the handgrip test which disabled analysis or adjustment for perfusion pressures. Future studies should include the simultaneous recording of IOP. Thirdly, the biasing effect of concomitant antihypertensive or topical medication use and the presence of arterial hypertension were not assessed.

## 5. Conclusion

We were able to validate baseline VD differences between glaucoma patients (NTG and POAG) and healthy controls. Additionally, NTG exhibited higher baseline peripapillary VDs than POAG [except for the inferotemporal and superotemporal regions, where focal rim thinning (notching) typically occurs]. Further, the importance of statistical correction for BP (MAP), gender and age in OCTA studies was proven. Future studies on OCTA should therefore correct for confounders as BP, gender and age (e.g., *via* adjusted linear mixed models). Finally, there was no significant VD change after isometric exercise in any of the groups. Further investigation with larger populations or other dynamic examination methods are recommended.

## Data availability statement

The raw data supporting the conclusions of this article will be made available by the authors, without undue reservation.

## Ethics statement

The studies involving human participants were reviewed and approved by the De Ethische Commissie Onderzoek UZ/KU Leuven (EC Onderzoek). The patients/participants provided their written informed consent to participate in this study.

## Author contributions

JVE, JBB, and IS analyzed and interpreted the data and wrote the manuscript. DDW, MD, and GM contributed to statistical guidance, and reviewed and approved the manuscript. CP and AH reviewed and approved the manuscript. JVE, JBB, IS, and AH designed the study. IS was the guarantor of the work and had full access to all the data in the study, takes responsibility for the integrity of the data, and the accuracy of the data analysis. All authors contributed to the article and approved the submitted version.

## References

[1] Tham YC Li X Wong TY Quigley HA Aung T Cheng CY. Global prevalence of glaucoma and projections of glaucoma burden through 2040: a systematic review and meta-analysis. *Ophthalmology.* (2014) 121:2081–90. 10.1016/j.ophtha.2014.05.013 24974815

[2] Esporcatte BL Tavares IM. Normal-tension glaucoma: an update. *Arq Bras Oftalmol.* (2016) 79:270–6. 10.5935/0004-2749.20160077 27626157

[3] Agnifili L Mastropasqua R Frezzotti P Fasanella V Motolese I Pedrotti E Circadian intraocular pressure patterns in healthy subjects, primary open angle and normal tension glaucoma patients with a contact lens sensor. *Acta Ophthalmol.* (2015) 93:e14–21. 10.1111/aos.12408 24720477

[4] Costagliola C Agnifili L Mastropasqua L di Costanzo A. Low-tension glaucoma: an oxymoron in ophthalmology. *Prevent Chronic Dis.* (2019) 16:E10. 10.5888/pcd16.180534 30676934 PMC6362709

[5] Vu DM Silva FQ Haseltine SJ Ehrlich JR Radcliffe NM. Relationship between corneal hysteresis and optic nerve parameters measured with spectral domain optical coherence tomography. *Graefes Arch Clin Exp Ophthalmol.* (2013) 251:1777–83. 10.1007/s00417-013-2311-x 23519885

[6] Hussnain SA Alsberge JB Ehrlich JR Shimmyo M Radcliffe NM. Change in corneal hysteresis over time in normal, glaucomatous and diabetic eyes. *Acta Ophthalmol.* (2015) 93:e627–30. 10.1111/aos.12726 25923367

[7] Barbosa-Breda J Van Keer K Abegão-Pinto L Nassiri V Molenberghs G Willekens K Improved discrimination between normal-tension and primary open-angle glaucoma with advanced vascular examinations - the Leuven Eye Study. *Acta Ophthalmol.* (2019) 97:e50–6. 10.1111/aos.13809 30225863

[8] Choi J Kook MS. Systemic and ocular hemodynamic risk factors in glaucoma. *Biomed Res Int.* (2015) 2015:141905. 10.1155/2015/141905 PMC4628774 26557650

[9] Lestak J Pitrova S Nutterova E Bartosova L. Normal tension vs high tension glaucoma: an - overview. *Cesk Slov Oftalmol Casopis Cesk Oftalmologicke Spolecnosti Slovenske Oftalmologicke Spolecnosti.* (2019) 75:55–60. 10.31348/2019/2/1 31537073

[10] Abegao Pinto L Willekens K Van Keer K Shibesh A Molenberghs G Vandewalle E Ocular blood flow in glaucoma - the Leuven Eye Study. *Acta Ophthalmol.* (2016) 94:592–8. 26895610 10.1111/aos.12962

[11] Lee SH Kim GA Lee W Bae HW Seong GJ Kim CY. Vascular and metabolic comorbidities in open-angle glaucoma with low- and high-teen intraocular pressure: a cross-sectional study from South Korea. *Acta Ophthalmol.* (2017) 95:e564–74. 10.1111/aos.13487 28677865

[12] Trivli A Koliarakis I Terzidou C Goulielmos GN Siganos CS Spandidos DA Normal-tension glaucoma: pathogenesis and genetics (Review). *Exp Ther Med.* (2019) 17:563–74. 30651837 10.3892/etm.2018.7011 PMC6307418

[13] Gugleta K Orgül S Hasler PW Picornell T Gherghel D Flammer J. Choroidal vascular reaction to hand-grip stress in subjects with vasospasm and its relevance in glaucoma. *Invest Ophthalmol Vis Sci.* (2003) 44:1573–80. 10.1167/iovs.02-0521 12657594

[14] Flammer J Orgül S Costa VP Orzalesi N Krieglstein GK Serra LM The impact of ocular blood flow in glaucoma. *Prog Retin Eye Res.* (2002) 21:359–93. 10.1016/S1350-9462(02)00008-3 12150988

[15] Van Melkebeke L Barbosa-Breda J Huygens M Stalmans I. Optical coherence tomography angiography in glaucoma: a review. *Ophthalmic Res.* (2018) 60:139–51. 10.1159/000488495 29794471

[16] Sousa DC Leal I Moreira S do Vale S Silva-Herdade AS Aguiar P A protocol to evaluate retinal vascular response using optical coherence tomography angiography. *Front Neurosci.* (2019) 13:566. 10.3389/fnins.2019.00566 31249500 PMC6582622

[17] Himori N Kunikata H Shiga Y Omodaka K Maruyama K Takahashi H The association between systemic oxidative stress and ocular blood flow in patients with normal-tension glaucoma. *Graefes Arch Clin Exp Ophthalmol.* (2016) 254:333–41. 26514963 10.1007/s00417-015-3203-z

[18] Yilmaz N Coban DT Bayindir A Erol MK Ellidag HY Giray O Higher serum lipids and oxidative stress in patients with normal tension glaucoma, but not pseudoexfoliative glaucoma. *Bosn J Basic Med Sci.* (2016) 16:21–7. 10.17305/bjbms.2016.830 26773174 PMC4765935

[19] Mroczkowska S Ekart A Sung V Negi A Qin L Patel SR Coexistence of macro- and micro-vascular abnormalities in newly diagnosed normal tension glaucoma patients. *Acta Ophthalmol.* (2012) 90:e553–9. 10.1111/j.1755-3768.2012.02494.x 22998650

[20] Melgarejo JD Eijgen JV Maestre GE Al-Aswad LA Thijs L Mena LJ Open-angle glaucomatous optic neuropathy is related to dips rather than increases in the mean arterial pressure over 24-H. *Am J Hypertens.* (2022) 35:703–14. 10.1093/ajh/hpac028 35218651 PMC9340631

[21] Mallick J Devi L Malik PK Mallick J. Update on normal tension glaucoma. *J Ophthalmic Vis Res.* (2016) 11:204–8. 10.4103/2008-322X.183914 27413503 PMC4926570

[22] Killer HE Pircher A. Normal tension glaucoma: review of current understanding and mechanisms of the pathogenesis. *Eye (Lond).* (2018) 32:924–30. 10.1038/s41433-018-0042-2 29456252 PMC5944657

[23] Fan N Wang P Tang L Liu X. Ocular blood flow and normal tension glaucoma. *Biomed Res Int.* (2015) 2015:308505. 10.1155/2015/308505 26558263 PMC4628977

[24] Hommer N Kallab M Sim YC Lee AX Chua J Tan B Effect of hyperoxia and hypoxia on retinal vascular parameters assessed with optical coherence tomography angiography. *Acta Ophthalmol.* (2022) 100:e1272–9. 10.1111/aos.15077 34881512

[25] Kallab M Hommer N Tan B Pfister M Schlatter A Werkmeister RM Plexus-specific effect of flicker-light stimulation on the retinal microvasculature assessed with optical coherence tomography angiography. *Am J Physiol Heart Circ Physiol.* (2021) 320:H23–8. 10.1152/ajpheart.00495.2020 33275537

[26] Nesper PL Lee HE Fayed AE Schwartz GW Yu F Fawzi AA. Hemodynamic response of the three macular capillary plexuses in dark adaptation and flicker stimulation using optical coherence tomography angiography. *Invest Ophthalmol Vis Sci.* (2019) 60:694–703. 10.1167/iovs.18-25478 30786274 PMC6383834

[27] Sousa DC Leal I Moreira S do Vale S Silva-Herdade AS Aguiar P Retinal vascular reactivity in Type 1 diabetes patients without retinopathy using optical coherence tomography angiography. *Invest Ophthalmol Vis Sci.* (2020) 61:49. 10.1167/iovs.61.6.49 32574352 PMC7415313

[28] Lommatzsch C Rothaus K Koch JM Heinz C Grisanti S. Vessel density in glaucoma of different entities as measured with optical coherence tomography angiography. *Clin Ophthalmol.* (2019) 13:2527–34. 31908407 10.2147/OPTH.S230192 PMC6925548

[29] Scripsema NK Garcia PM Bavier RD Chui TY Krawitz BD Mo S Optical coherence tomography angiography analysis of perfused peripapillary capillaries in primary open-angle glaucoma and normal-tension glaucoma. *Invest Ophthalmol Vis Sci.* (2016) 57:OCT611–20. 10.1167/iovs.15-18945 27742922

[30] Hosari S Hohberger B Theelke L Sari H Lucio M Mardin CYOCT. Angiography: measurement of retinal macular microvasculature with spectralis II OCT angiography - reliability and reproducibility. *Ophthalmologica.* (2020) 243:75–84. 10.1159/000502458 31509842

[31] Bojikian KD Chen CL Wen JC Zhang Q Xin C Gupta D Optic disc perfusion in primary open angle and normal tension glaucoma eyes using optical coherence tomography-based microangiography. *PLoS One.* (2016) 11:e0154691. 10.1371/journal.pone.0154691 27149261 PMC4858256

[32] Wang YM Shen R Lin TPH Chan PP Wong MOM Chan NCY Optical coherence tomography angiography metrics predict normal tension glaucoma progression. *Acta Ophthalmol.* (2022) 100:e1455–62. 10.1111/aos.15117 35261173

[33] Ko CK Huang KI Su FY Ko ML. Vessel density in the macular and peripapillary areas in preperimetric glaucoma to various stages of primary open-angle glaucoma in Taiwan. *J Clin Med.* (2021) 10:5490. 10.3390/jcm10235490 34884191 PMC8658219

[34] Wang X Jiang C Ko T Kong X Yu X Min W Correlation between optic disc perfusion and glaucomatous severity in patients with open-angle glaucoma: an optical coherence tomography angiography study. *Graefes Arch Clin Exp Ophthalmol.* (2015) 253:1557–64. 26255817 10.1007/s00417-015-3095-y

[35] Miguel A Silva A Barbosa-Breda J Azevedo L Abdulrahman A Hereth E OCT-angiography detects longitudinal microvascular changes in glaucoma: a systematic review. *Br J Ophthalmol.* (2021) 106:667–75. 10.1136/bjophthalmol-2020-318166 33452184

[36] Shin JW Sung KR Lee JY Kwon J Seong M. Optical coherence tomography angiography vessel density mapping at various retinal layers in healthy and normal tension glaucoma eyes. *Graefes Arch Clin Exp Ophthalmol.* (2017) 255:1193–202. 10.1007/s00417-017-3671-4 28429123

[37] Xu H Zhai R Zong Y Kong X Jiang C Sun X Comparison of retinal microvascular changes in eyes with high-tension glaucoma or normal-tension glaucoma: a quantitative optic coherence tomography angiographic study. *Graefes Arch Clin Exp Ophthalmol.* (2018) 256:1179–86. 10.1007/s00417-018-3930-z 29450622

[38] Lin Y Jiang H Liu Y Rosa Gameiro G Gregori G Dong C Age-related alterations in retinal tissue perfusion and volumetric vessel density. *Invest Ophthalmol Vis Sci.* (2019) 60:685–93. 10.1167/iovs.18-25864 30786280 PMC6383727

[39] Blum M Bachmann K Wintzer D Riemer T Vilser W Strobel J. Noninvasive measurement of the Bayliss effect in retinal autoregulation. *Graefes Arch Clin Exp Ophthalmol.* (1999) 237:296–300. 10208262 10.1007/s004170050236

[40] Köchli S Endes K Infanger D Zahner L Hanssen H. Obesity, blood pressure, and retinal vessels: a meta-analysis. *Pediatrics.* (2018) 141:e20174090. 10.1542/peds.2017-4090 29743194

[41] Konukoglu D Uzun H. Endothelial dysfunction and hypertension. *Adv Exp Med Biol.* (2017) 956:511–40. 28035582 10.1007/5584_2016_90

[42] Chou WY Liu CJ Chen MJ Chiou SH Chen WT Ko YC. Effect of cold provocation on vessel density in eyes with primary open angle glaucoma: an optical coherence tomography angiography study. *Sci Rep.* (2019) 9:9384. 10.1038/s41598-019-45386-7 31253843 PMC6599001

[43] Gherghel D Hosking SL Cunliffe IA. Abnormal systemic and ocular vascular response to temperature provocation in primary open-angle glaucoma patients: a case for autonomic failure? *Invest Ophthalmol Vis Sci.* (2004) 45:3546–54. 10.1167/iovs.04-0290 15452061

[44] Zhu J Merkle CW Bernucci MT Chong SP Srinivasan VJ. Can OCT angiography be made a quantitative blood measurement tool? *Appl Sci (Basel).* (2017) 7:687. 10.3390/app7070687 30009045 PMC6042878

[45] Streese L Lona G Wagner J Knaier R Burri A Nève G Normative data and standard operating procedures for static and dynamic retinal vessel analysis as biomarker for cardiovascular risk. *Sci Rep.* (2021) 11:14136. 10.1038/s41598-021-93617-7 34238996 PMC8266855

[46] Gugleta K Kochkorov A Waldmann N Polunina A Katamay R Flammer J Dynamics of retinal vessel response to flicker light in glaucoma patients and ocular hypertensives. *Graefes Arch Clin Exp Ophthalmol.* (2012) 250:589–94. 10.1007/s00417-011-1842-2 22008947

[47] Selbach JM Schallenberg M Kramer S Anastassiou G Steuhl KP Vilser W Trabeculectomy improves vessel response measured by dynamic vessel analysis (DVA) in glaucoma patients. *Open Ophthalmol J.* (2014) 8:75–81. 10.2174/1874364101408010075 25352934 PMC4209500

