# Supplementary material for: Normal tension glaucoma: A dynamic optical coherence tomography angiography study
Source: Front Med (Lausanne). 2023 Jan 6;9:1037471. doi: 10.3389/fmed.2022.1037471 (PMC9853195; doi:10.3389/fmed.2022.1037471)
Supplement: Supplementary file 1 [file Data_Sheet_1.docx]

**SUPPLEMENTARY MATERIALS**

**Table S1. Summary of IOP-lowering medication and preceding surgery of the glaucoma subgroups.**

| **Diagnosis** | **Single BB** | **Single CAI** | **Single PG** | **Single AA** | **Fix Bb+PG** | **Fix Bb+AA** | **Fix BB+CAI** | **Trab** | **Tube** | **Phaco** | **ALT** |
| --- | --- | --- | --- | --- | --- | --- | --- | --- | --- | --- | --- |
| NTG | 0 | 0 | 1 | 0 | 0 | 1 | 0 | 1 | 0 | 0 | 0 |
| NTG | 0 | 1 | 1 | 1 | 0 | 0 | 0 | 1 | 0 | 0 | 0 |
| NTG | 0 | 0 | 1 | 0 | 0 | 0 | 1 | 0 | 0 | 0 | 0 |
| NTG | 0 | 0 | 1 | 0 | 0 | 0 | 1 | 0 | 0 | 0 | 0 |
| NTG | 1 | 0 | 0 | 0 | 0 | 0 | 0 | 0 | 0 | 0 | 0 |
| NTG | 0 | 0 | 1 | 0 | 0 | 0 | 0 | 0 | 0 | 0 | 0 |
| NTG | 0 | 1 | 1 | 0 | 0 | 0 | 0 | 0 | 0 | 0 | 0 |
| NTG | 0 | 0 | 0 | 0 | 0 | 0 | 0 | 1 | 0 | 0 | 0 |
| NTG | 0 | 0 | 1 | 0 | 0 | 0 | 1 | 1 | 0 | 0 | 0 |
| NTG | 0 | 0 | 1 | 0 | 0 | 0 | 1 | 1 | 0 | 0 | 0 |
| NTG | 0 | 0 | 1 | 0 | 0 | 0 | 1 | 1 | 0 | 0 | 0 |
| NTG | 0 | 0 | 1 | 0 | 0 | 0 | 1 | 1 | 0 | 0 | 0 |
| NTG | 1 | 0 | 0 | 0 | 0 | 0 | 0 | 1 | 0 | 0 | 0 |
| NTG | 0 | 0 | 0 | 0 | 0 | 1 | 0 | 0 | 0 | 0 | 0 |
| NTG | 0 | 0 | 0 | 0 | 0 | 0 | 0 | 0 | 0 | 0 | 0 |
| NTG | 0 | 1 | 1 | 0 | 0 | 0 | 0 | 0 | 0 | 0 | 0 |
| **NTG Total** | **2** | **3** | **11** | **1** | **0** | **2** | **6** | **8** | **0** | **0** | **0** |
| POAG | 0 | 0 | 1 | 0 | 0 | 0 | 0 | 0 | 0 | 1 | 0 |
| POAG | 1 | 0 | 0 | 0 | 0 | 0 | 0 | 0 | 0 | 1 | 0 |
| POAG | 0 | 0 | 1 | 0 | 0 | 0 | 1 | 0 | 0 | 0 | 0 |
| POAG | 0 | 1 | 1 | 0 | 0 | 0 | 0 | 0 | 0 | 1 | 0 |
| POAG | 0 | 1 | 0 | 0 | 1 | 0 | 0 | 1 | 0 | 0 | 0 |
| POAG | 0 | 0 | 0 | 0 | 1 | 0 | 0 | 0 | 0 | 0 | 0 |
| POAG | 0 | 0 | 0 | 0 | 0 | 0 | 0 | 1 | 0 | 0 | 0 |
| POAG | 0 | 0 | 1 | 0 | 0 | 1 | 0 | 1 | 0 | 0 | 0 |
| POAG | 0 | 0 | 1 | 0 | 0 | 1 | 0 | 1 | 0 | 0 | 0 |
| POAG | 0 | 0 | 0 | 0 | 1 | 0 | 0 | 0 | 0 | 0 | 1 |
| POAG | 0 | 0 | 0 | 0 | 0 | 0 | 1 | 1 | 0 | 0 | 0 |
| POAG | 0 | 0 | 0 | 0 | 1 | 0 | 1 | 1 | 1 | 0 | 1 |
| **POAG Total** | **1** | **2** | **5** | **0** | **4** | **2** | **3** | **6** | **1** | **3** | **2** |

POAG: Primary open angle glaucoma; NTG: Normal tension glaucoma; BB: Beta blocker; CAI: Carbonic anhydrase inhibitors; PG: prostaglandin; AA: Alpha-adrenergic agonist; Fix: fixed combination of two medication classes; Trab: trabeculectomy; Phaco: phaco-emulsification; ALT: Argon Laser Trabeculoplasty.

**Table S2. Optic nerve head mean vessel density (%, OCTA) and standard deviation.**

| **Diagnosis** | **Time** |  | **All** | **Disc** | **Peripapil** | **Sup Hemi** | **Inf Hemi** | **NS** | **NI** | **IN** | **IT** | **TI** | **TS** | **ST** | **SN** |
| --- | --- | --- | --- | --- | --- | --- | --- | --- | --- | --- | --- | --- | --- | --- | --- |
| **NTG** | **Baseline** | **Mean** | 46.9 | 54.7 | 48.5 | 50.1 | 45.7 | 42.4 | 40.4 | 36.6 | 38.2 | 45.8 | 49.4 | 41.5 | 41.3 |
|  |  | **Std Dev** | 7.0 | 9.6 | 8.5 | 9.1 | 9.4 | 9.2 | 11.0 | 12.2 | 13.6 | 7.4 | 9.1 | 14.5 | 11.3 |
|  | **During** | **Mean** | 46.0 | 54.5 | 46.8 | 48.2 | 45.3 | 41.3 | 40.2 | 36.8 | 36.9 | 45.8 | 48.8 | 39.6 | 40.7 |
|  |  | **Std Dev** | 6.0 | 8.3 | 7.9 | 8.4 | 8.1 | 10.4 | 10.3 | 10.6 | 14.8 | 7.9 | 8.5 | 13.2 | 10.4 |
| **POAG** | **Baseline** | **Mean** | 42.5 | 58.6 | 42.6 | 44.4 | 41.7 | 36.7 | 35.1 | 33.7 | 32.7 | 42.0 | 43.5 | 36.0 | 32.1 |
|  |  | **Std Dev** | 5.8 | 6.3 | 7.1 | 7.8 | 6.9 | 7.4 | 5.7 | 9.6 | 14.7 | 6.5 | 10.8 | 13.8 | 9.9 |
|  | **During** | **Mean** | 42.2 | 56.9 | 42.7 | 43.6 | 41.7 | 35.7 | 33.9 | 32.5 | 33.4 | 42.4 | 44.8 | 34.7 | 31.4 |
|  |  | **Std Dev** | 5.3 | 4.9 | 6.7 | 7.3 | 7.0 | 5.7 | 6.9 | 10.9 | 14.2 | 5.5 | 7.5 | 14.7 | 10.3 |
| **Healthy** | **Baseline** | **Mean** | 55.9 | 59.2 | 58.1 | 58.2 | 58.1 | 50.1 | 48.5 | 51.5 | 59.1 | 50.1 | 53.7 | 55.2 | 47.6 |
|  |  | **Std Dev** | 2.1 | 4.1 | 2.3 | 2.3 | 2.8 | 3.5 | 3.2 | 4.8 | 4.8 | 4.3 | 5.0 | 5.5 | 4.6 |
|  | **During** | **Mean** | 56.0 | 59.8 | 58.4 | 58.5 | 58.4 | 50.7 | 48.6 | 51.7 | 59.4 | 51.1 | 54.4 | 54.6 | 47.9 |
|  |  | **Std Dev** | 2.4 | 5.3 | 2.8 | 3.0 | 2.7 | 2.7 | 4.0 | 4.4 | 4.8 | 4.2 | 4.5 | 6.7 | 5.4 |

NTG: Normal tension glaucoma POAG: Primary open angel glaucoma; Peripapil: peripapillary mean; SUP: superior; Hemi: hemisphere. INF: inferior; NS NI IN IT TI TS ST SN as explained in the Materials and Methods section.

**Table S3. Macular superficial and deep vascular plexus mean vessel density (%, OCTA) and standard deviation.**

| **Diagnosis** | **Time** |  | **All** | **All SUP Hemi** | **All INF Hemi** | **FAZ Area** | **Fovea** | **ParaFovea** | **Para SUP Hemi** | **Para INF Hemi** | **Para SUP** | **Para INF** | **Para NAS** | **Para TEMP** | **PeriFovea** | **Peri SUP Hemi** | **Peri INF Hemi** | **Peri SUP** | **Peri INF** | **Peri NAS** | **Peri TEMP** |
| --- | --- | --- | --- | --- | --- | --- | --- | --- | --- | --- | --- | --- | --- | --- | --- | --- | --- | --- | --- | --- | --- |
|  |  |  |  |  |  |  |  | **Macular superficial vascular plexus (ILM-IPL)** | | | | | | | |  |  |  |  |  |  |
| **NTG** | **Baseline** | **Mean** | 38.8 | 39.3 | 38.2 | 0.2 | 19.2 | 42.3 | 43.1 | 41.4 | 43.0 | 41.0 | 43.4 | 41.7 | 38.8 | 39.7 | 38.0 | 38.3 | 37.2 | 44.2 | 35.6 |
|  |  | **Std Dev** | 6.0 | 6.4 | 6.3 | 0.1 | 5.5 | 5.7 | 7.0 | 6.1 | 10.2 | 7.1 | 6.0 | 6.3 | 6.5 | 7.0 | 6.7 | 8.3 | 7.5 | 6.4 | 6.4 |
|  | **During** | **Mean** | 37.9 | 38.5 | 37.2 | 0.3 | 18.1 | 41.1 | 42.2 | 40.0 | 43.8 | 40.0 | 40.9 | 39.9 | 37.8 | 38.7 | 36.9 | 37.7 | 35.5 | 43.1 | 34.9 |
|  |  | **Std Dev** | 5.6 | 5.9 | 5.7 | 0.1 | 6.4 | 5.6 | 6.5 | 5.9 | 6.7 | 7.1 | 7.5 | 6.1 | 6.1 | 6.8 | 5.9 | 8.5 | 6.1 | 5.9 | 5.9 |
| **POAG** | **Baseline** | **Mean** | 38.7 | 39.2 | 37.7 | 0.3 | 19.0 | 42.7 | 42.9 | 42.5 | 44.0 | 42.8 | 42.5 | 41.5 | 38.5 | 39.2 | 38.2 | 37.1 | 37.3 | 44.0 | 35.4 |
|  |  | **Std Dev** | 5.3 | 5.1 | 6.3 | 0.1 | 6.2 | 6.9 | 6.9 | 7.2 | 6.2 | 7.9 | 7.8 | 7.4 | 5.3 | 5.2 | 6.5 | 5.7 | 7.4 | 6.2 | 5.3 |
|  | **During** | **Mean** | 37.1 | 37.8 | 36.4 | 0.3 | 18.0 | 41.7 | 42.6 | 40.9 | 43.9 | 41.1 | 42.9 | 39.1 | 36.9 | 37.6 | 36.2 | 35.4 | 34.8 | 42.7 | 34.6 |
|  |  | **Std Dev** | 5.7 | 5.3 | 6.5 | 0.1 | 6.3 | 6.4 | 6.4 | 6.7 | 5.0 | 7.2 | 6.6 | 9.1 | 5.8 | 5.4 | 6.7 | 5.8 | 7.4 | 6.2 | 5.5 |
| **Healthy** | **Baseline** | **Mean** | 49.8 | 49.4 | 50.2 | 0.3 | 19.3 | 52.1 | 51.9 | 52.4 | 53.0 | 53.0 | 50.7 | 51.9 | 50.2 | 49.9 | 50.5 | 49.9 | 50.4 | 54.4 | 46.1 |
|  |  | **Std Dev** | 3.9 | 4.3 | 3.6 | 0.1 | 5.2 | 4.5 | 4.5 | 4.7 | 5.1 | 4.7 | 5.0 | 4.3 | 4.3 | 4.9 | 4.0 | 5.3 | 4.5 | 4.1 | 4.4 |
|  | **During** | **Mean** | 48.2 | 48.3 | 48.2 | 0.3 | 19.2 | 50.8 | 50.9 | 50.8 | 51.1 | 52.1 | 49.8 | 50.3 | 48.5 | 48.5 | 48.6 | 48.6 | 48.2 | 52.9 | 44.4 |
|  |  | **Std Dev** | 3.2 | 3.2 | 3.5 | 0.1 | 6.1 | 4.0 | 4.2 | 4.2 | 5.1 | 4.3 | 5.1 | 4.0 | 3.3 | 3.3 | 3.5 | 3.4 | 3.9 | 3.6 | 3.8 |
|  |  |  |  |  |  |  |  | **Macular deep vascular plexus (IPL-OPL)** | | | | | | | | | |  |  |  |  |
| **NTG** | **Baseline** | **Mean** | 44.7 | 44.5 | 44.9 |  | 35.2 | 49.8 | 49.5 | 50.1 | 47.7 | 49.0 | 50.7 | 51.9 | 45.4 | 45.7 | 45.1 | 44.3 | 43.9 | 45.8 | 47.6 |
|  |  | **Std Dev** | 6.7 | 7.1 | 6.5 |  | 6.7 | 6.1 | 7.9 | 5.5 | 9.7 | 6.2 | 8.1 | 5.9 | 7.3 | 7.6 | 7.3 | 7.8 | 8.1 | 7.3 | 7.7 |
|  | **During** | **Mean** | 42.8 | 43.3 | 42.3 |  | 33.5 | 48.3 | 49.0 | 47.6 | 48.3 | 46.2 | 48.8 | 49.8 | 43.2 | 44.2 | 42.2 | 42.8 | 40.1 | 44.1 | 45.9 |
|  |  | **Std Dev** | 6.1 | 6.1 | 6.6 |  | 8.3 | 5.0 | 5.4 | 5.5 | 6.2 | 7.9 | 5.6 | 5.5 | 6.9 | 7.0 | 7.3 | 7.9 | 8.1 | 7.0 | 7.5 |
| **POAG** | **Baseline** | **Mean** | 45.9 | 46.3 | 45.1 |  | 35.3 | 51.9 | 52.4 | 51.4 | 51.5 | 50.3 | 52.1 | 53.7 | 46.3 | 47.0 | 46.5 | 45.3 | 45.9 | 47.1 | 47.3 |
|  |  | **Std Dev** | 5.8 | 6.3 | 6.3 |  | 9.2 | 4.7 | 4.6 | 5.0 | 5.2 | 5.7 | 5.4 | 4.2 | 6.5 | 6.9 | 6.6 | 8.0 | 7.2 | 6.8 | 6.7 |
|  | **During** | **Mean** | 44.9 | 45.5 | 44.3 |  | 35.5 | 51.3 | 52.8 | 49.7 | 52.1 | 48.3 | 53.3 | 51.3 | 45.4 | 45.9 | 44.9 | 44.0 | 43.3 | 46.7 | 47.5 |
|  |  | **Std Dev** | 7.6 | 7.0 | 8.3 |  | 6.8 | 5.5 | 4.7 | 6.9 | 5.9 | 7.8 | 4.7 | 8.7 | 8.5 | 8.0 | 9.2 | 8.7 | 10.0 | 9.2 | 7.9 |
| **Healthy** | **Baseline** | **Mean** | 50.8 | 50.6 | 50.9 |  | 35.8 | 54.8 | 55.0 | 54.6 | 54.3 | 53.3 | 55.3 | 56.3 | 52.1 | 52.1 | 52.1 | 51.3 | 50.8 | 52.0 | 54.1 |
|  |  | **Std Dev** | 5.4 | 5.6 | 5.4 |  | 5.4 | 3.3 | 3.5 | 3.2 | 4.1 | 3.9 | 3.5 | 3.3 | 6.1 | 6.2 | 6.3 | 6.1 | 7.3 | 7.6 | 5.1 |
|  | **During** | **Mean** | 48.1 | 48.0 | 48.2 |  | 36.0 | 52.4 | 52.2 | 52.6 | 50.9 | 52.0 | 52.9 | 53.8 | 49.4 | 49.4 | 49.4 | 48.5 | 48.4 | 48.5 | 52.2 |
|  |  | **Std Dev** | 5.1 | 5.3 | 5.1 |  | 8.1 | 4.0 | 4.2 | 3.9 | 5.2 | 4.6 | 4.6 | 4.2 | 5.5 | 5.6 | 5.7 | 5.9 | 6.4 | 6.5 | 4.6 |

**Table S4. Optic nerve head vessel density (OCTA) adjusted linear mixed model p-values.**

NTG: Normal tension glaucoma; POAG: Primary open angel glaucoma; NTG: Normal tension glaucoma; SUP: superior; Hemi: hemisphere; INF: inferior; FAZ: foveal avascular zone; Para: parafovea; NAS: nasal; TEM: temporal.

| **VD region** | **All** | **Disc** | **Peripapil** | **Sup Hemi** | **Inf Hemi** | **NS** | **NI** | **IN** | **IT** | **TI** | **TS** | **ST** | **SN** |
| --- | --- | --- | --- | --- | --- | --- | --- | --- | --- | --- | --- | --- | --- |
| **Intercept** | <0.001 | <0.001 | <0.001 | <0.001 | 0.0004 | 0.012 | 0.008 | 0.012 | 0.005 | <0.001 | <0.001 | 0.007 | 0.001 |
| **Gender** |  |  | 0.020 | 0.039 | 0.025 | 0.010 |  |  |  |  |  |  |  |
| **MAP_0** |  |  |  |  |  |  |  |  |  |  |  |  |  |
| **Age** |  |  |  |  |  |  |  |  |  |  |  |  |  |
| **Diagnosis** |  |  |  |  |  |  |  |  |  |  |  |  |  |
| POAG (vs normal) | <0.001 |  | <0.001 | <0.001 | <0.001 | <0.001 | <0.001 | <0.001 | <0.001 | 0.001 | 0.003 | <0.001 | <0.001 |
| NTG (vs normal) | <0.001 |  | <0.001 | <0.001 | <0.001 | 0.003 | 0.005 | <0.001 | <0.001 |  |  | 0.002 | 0.049 |
| NTG (vs POAG) | 0.024 |  | 0.035 | 0.043 |  | 0.025 |  |  |  | 0.049 | 0.034 |  | 0.006 |
| **Time** |  |  |  |  |  |  |  |  |  |  |  |  |  |
| **Gender*time** |  |  |  |  |  |  |  |  |  |  | 0.048 |  |  |
| **MAP_0*time** |  |  |  |  |  |  |  |  |  |  |  |  |  |
| **MAPdiff*time** |  |  |  |  |  |  |  |  |  |  |  |  |  |
| **Age*time** |  |  |  |  |  |  |  |  |  |  |  |  |  |
| **Diagnosis*time** |  |  |  |  |  |  |  |  |  |  |  |  |  |
| POAG (vs normal) |  |  |  |  |  |  |  |  |  |  |  |  |  |
| NTG (vs normal) |  |  |  |  |  |  |  |  |  |  |  |  |  |
| NTG (vs POAG) |  |  |  |  |  |  |  |  |  |  |  |  |  |

Only significant responses are reported. VD: Vessel Density; MAP_0: mean arterial pressure at baseline; POAG: Primary open angel glaucoma; NTG: Normal tension glaucoma; MAPdiff: difference of mean arterial pressure during examination; Peripapil: peripapillary mean; SUP: superior; Hemi: hemisphere. INF: inferior; NS NI IN IT TI TS ST SN as explained in the Materials and Methods section.**Table S5. Macular superficial (ILM-IPL) vascular plexus vessel density (OCTA) adjusted linear mixed model p-values.**

| **Vessel Density Region (ETDRS)** | **All** | **All SUP Hemi** | **All INF Hemi** | **FAZ Area** | **Fovea** | **Para**  **Fovea** | **Para SUP Hemi** | **Para INF Hemi** | **Para SUP** | **Para INF** | **Para NAS** | **Para TEMP** | **Peri**  **Fovea** | **Peri SUP Hemi** | **Peri INF Hemi** | **Peri SUP** | **Peri INF** | **Peri NAS** | **Peri TEMP** |
| --- | --- | --- | --- | --- | --- | --- | --- | --- | --- | --- | --- | --- | --- | --- | --- | --- | --- | --- | --- |
| **Intercept** | <0.001 | <0.001 | <0.001 | 0.0148 | 0.0447 | <0.001 | <0.001 | <0.001 | <0.001 | <0.001 | <0.001 | <0.001 | <0.001 | <0.001 | <0.001 | <0.001 | <0.001 | <0.001 | <0.001 |
| **Gender** |  |  |  |  |  |  |  |  |  |  |  |  |  |  |  |  |  |  |  |
| **MAP_0** |  |  |  |  |  |  |  |  |  |  |  |  |  |  |  |  |  |  |  |
| **Age** |  | 0.037 |  |  |  |  |  |  | 0.039 |  |  |  |  | 0.041 |  | 0.044 |  | 0.016 |  |
| **Diagnosis** |  |  |  |  |  |  |  |  |  |  |  |  |  |  |  |  |  |  |  |
| POAG (vs normal) | <0.001 | <0.001 | <0.001 |  |  | <0.001 | 0.002 | <0.001 | 0.003 | 0.001 | 0.009 | <0.001 | <0.001 | <0.001 | <0.001 | <0.001 | <0.001 | <0.001 | <0.001 |
| NTG (vs normal) | <0.001 | <0.001 | <0.001 |  |  | <0.001 | <0.001 | <0.001 | <0.001 | <0.001 | 0.0131 | <0.001 | <0.001 | <0.001 | <0.001 | <0.001 | <0.001 | <0.001 | <0.001 |
| NTG (vs POAG) |  |  |  |  |  |  |  |  |  |  |  |  |  |  |  |  |  |  |  |
| **Time** |  |  |  |  |  |  |  |  |  |  |  |  |  |  |  |  |  |  |  |
| **Gender*time** |  |  |  |  |  | 0.010 | 0.028 | 0.015 |  | 0.048 | 0.014 |  |  |  |  |  |  |  |  |
| **MAP_0*time** |  |  |  |  |  | 0.003 | 0.022 | 0.002 | 0.017 | 0.008 | 0.020 |  |  |  |  |  |  |  |  |
| **MAPdiff*time** |  |  |  | 0.010 |  |  |  |  |  |  |  |  |  |  |  |  |  |  |  |
| **Age*time** |  |  |  |  | 0.035 |  |  |  |  |  |  |  |  |  |  |  |  |  |  |
| **Diagnosis*time** |  |  |  |  |  |  |  |  |  |  |  |  |  |  |  |  |  |  |  |
| POAG (vs normal) |  |  |  |  |  |  |  |  |  |  |  |  |  |  |  |  |  |  |  |
| NTG (vs normal) |  |  |  |  |  |  |  |  |  |  |  |  |  |  |  |  |  |  |  |
| NTG (vs POAG) |  |  |  |  |  |  |  |  |  |  |  |  |  |  |  |  |  |  |  |

Only significant responses are reported. MAP_0: mean arterial pressure at baseline; POAG: Primary open angel glaucoma; NTG: Normal tension glaucoma; MAPdiff: difference of mean arterial pressure during examination; SUP: superior; Hemi: hemisphere; INF: inferior; FAZ: foveal avascular zone; Para: parafovea; NAS: nasal; TEM: temporal.

**Table S6. Macular deep (IPL-OPL) vascular plexus vessel density (OCTA) adjusted linear mixed model p-values.**

| **Vessel Density Region (ETDRS)** | **All** | **All SUP Hemi** | **All INF Hemi** | **Fovea** | **Para**  **Fovea** | **Para SUP Hemi** | **Para INF Hemi** | **Para SUP** | **Para INF** | **Para NAS** | **Para TEMP** | **Peri**  **Fovea** | **Peri SUP Hemi** | **Peri INF Hemi** | **Peri SUP** | **Peri INF** | **Peri NAS** | **Peri TEMP** |
| --- | --- | --- | --- | --- | --- | --- | --- | --- | --- | --- | --- | --- | --- | --- | --- | --- | --- | --- |
| **Intercept** | <0.001 | <0.001 | <0.001 | 0.003 | <0.001 | <0.001 | <0.001 | <0.001 | <0.001 | <0.001 | <0.001 | <0.001 | <0.001 | <0.001 | <0.001 | <0.001 | <0.001 | <0.001 |
| **Gender** |  |  |  |  |  |  |  |  |  |  |  |  |  |  |  |  |  |  |
| **MAP_0** |  |  |  |  |  |  |  |  |  | 0.01 |  |  |  |  |  |  |  |  |
| **Age** |  |  |  |  |  |  |  |  |  | 0.044 |  |  |  |  |  |  |  |  |
| **Diagnosis** |  |  |  |  |  |  |  |  |  |  |  |  |  |  |  |  |  |  |
| POAG (vs normal) |  |  |  |  |  |  |  |  |  |  |  |  |  |  | 0.042 |  |  | 0.028 |
| NTG (vs normal) | 0.023 | 0.036 | 0.018 |  | 0.035 | 0.044 |  | 0.036 |  |  |  | 0.021 | 0.042 | 0.013 | 0.038 | 0.023 |  | 0.012 |
| NTG (vs POAG) |  |  |  |  |  |  |  |  |  |  |  |  |  |  |  |  |  |  |
| **Time** |  |  |  | 0.025 |  |  |  |  |  |  |  |  |  |  |  |  |  |  |
| **Gender*time** |  |  |  |  |  |  |  |  |  |  |  |  |  |  |  |  |  |  |
| **MAP_0*time** | 0.004 | 0.004 | 0.001 |  | <0.001 | 0.002 | <0.001 | 0.004 | 0.003 | 0.006 | 0.012 | 0.006 | 0.005 | 0.012 | 0.020 | 0.022 | 0.002 | 0.021 |
| **MAPdiff*time** |  |  |  |  |  |  |  |  |  |  |  |  |  |  |  |  |  |  |
| **Age*time** | <0.001 | <0.001 | <0.001 | <0.001 | <0.001 | 0.002 | <0.001 | 0.006 | <0.001 |  | 0.002 | <0.001 | <0.001 | <0.001 | 0.002 | <0.001 | <0.001 | 0.003 |
| **Diagnosis*time** |  |  |  |  |  |  |  |  |  |  |  |  |  |  |  |  |  |  |
| POAG (vs normal) |  |  |  |  |  |  |  |  |  |  |  |  |  |  |  |  |  |  |
| NTG (vs normal) |  |  |  | 0.012 |  |  |  |  | 0.016 | 0.042 |  |  |  |  |  |  |  |  |
| NTG (vs POAG) |  |  |  | 0.008 |  |  |  |  |  |  |  |  |  |  |  |  |  |  |

Only significant responses are reported. MAP_0: mean arterial pressure at baseline; POAG: Primary open angel glaucoma; NTG: Normal tension glaucoma; MAPdiff: difference of mean arterial pressure during examination; SUP: superior; Hemi: hemisphere; INF: inferior; Para: parafovea; NAS: nasal; TEM: temporal.

**Table S7. Optic nerve head total retinal thickness (OCT) adjusted linear mixed model estimates (µm).**

| **Region** | **Peripapillary** | **SUP Hemi** | **INF Hemi** | **TEM** | **SUP** | **NAS** | **INF** | **TS** | **ST** | **SN** | **NS** | **NI** | **IN** | **IT** | **TI** |
| --- | --- | --- | --- | --- | --- | --- | --- | --- | --- | --- | --- | --- | --- | --- | --- |
| **Intercept** | 132.4 | 123.9 | 146.1 | 79.5 | 153.7 | 106.4 | 207.9 | 86.8 | 191.0 | 120.4 | 116.9 | 95.4 | 203.6 | 215.1 | 70.9 |
| **Gender** |  |  |  |  |  |  |  |  |  |  |  |  |  |  |  |
| **MAP_0** |  |  |  |  |  |  |  |  |  |  |  |  |  |  |  |
| **Age** |  |  |  |  |  |  |  | -0.6 |  |  |  |  |  |  |  |
| **Diagnosis** |  |  |  |  |  |  |  |  |  |  |  |  |  |  |  |
| POAG (vs normal) | -41.2 | -21.7 | -44.6 | -8.8 | -49.9 | -37.1 | -69.3 | -10.6 | -43.7 | -55.1 | -40.4 | -31.5 | -72.6 | -65.1 |  |
| NTG (vs normal) | -27.6 | -37.3 | -32.4 |  | -32.0 | -19.9 | -55.5 |  | -36.7 | -29.1 | -22.2 | -16.9 | -51.5 | -59.9 |  |
| NTG (vs POAG) | 13.6 | 15.6 |  |  | 17.9 | 17.2 |  | 11.5 |  | 26.0 | 18.2 |  |  |  |  |
| **Time** |  |  |  |  |  |  |  |  |  |  |  |  |  |  |  |
| **Gender*time** |  |  |  |  |  |  |  |  |  |  |  |  |  |  |  |
| **MAP_0*time** |  |  |  | -0.1 |  |  |  |  |  |  |  |  |  |  |  |
| **MAPdiff*time** |  |  |  |  |  |  |  |  |  |  |  |  |  |  |  |
| **Age*time** |  |  |  |  |  |  |  |  |  |  |  |  |  |  |  |
| **Diagnosis*time** |  |  |  |  |  |  |  |  |  |  |  |  |  |  |  |
| POAG (vs normal) |  |  |  |  |  |  |  |  |  |  |  |  |  | 0.0 |  |
| NTG (vs normal) |  |  |  | -3.3 |  |  |  | -5.8 |  |  |  |  |  |  |  |
| NTG (vs POAG) | -3.1 |  |  | -3.4 |  |  |  |  |  |  |  |  |  | -4.9 |  |

Only significant responses are reported. MAP_0: mean arterial pressure at baseline; POAG: Primary open angel glaucoma; NTG: Normal tension glaucoma; MAPdiff: difference of mean arterial pressure during examination; Peripapil: peripapillary mean; SUP: superior; Hemi: hemisphere. INF: inferior; TEM: temporal; NAS: nasal; NS NI IN IT TI TS ST SN as explained in the Materials and Methods section.

**Table S8. Optic nerve head total retinal thickness (OCT) adjusted linear mixed model p-values.**

| **Region** | **Peripapillary** | **SUP Hemi** | **INF Hemi** | **TEM** | **SUP** | **NAS** | **INF** | **TS** | **ST** | **SN** | **NS** | **NI** | **IN** | **IT** | **TI** |
| --- | --- | --- | --- | --- | --- | --- | --- | --- | --- | --- | --- | --- | --- | --- | --- |
| **Intercept** | <0.001 | <0.001 | <0.001 | <0.001 | <0.001 | 0.006 | <0.001 | 0.001 | <0.001 | 0.017 | 0.004 | 0.023 | <0.001 | <0.001 | <0.001 |
| **Gender** |  |  |  |  |  |  |  |  |  |  |  |  |  |  |  |
| **MAP_0** |  |  |  |  |  |  |  |  |  |  |  |  |  | 0.024 |  |
| **Age** |  |  |  |  |  |  |  | 0.049 |  |  |  |  |  |  |  |
| **Diagnosis** |  |  |  |  |  |  |  |  |  |  |  |  |  |  |  |
| POAG (vs normal) | <0.001 | <0.001 | <0.001 | 0.039 | <0.001 | <0.001 | <0.001 | 0.047 | <0.001 | <0.001 | <0.001 | <0.001 | <0.001 | <0.001 |  |
| NTG (vs normal) | <0.001 | 0.002 | <0.001 |  | <0.001 | 0.008 | <0.001 |  | <0.001 | 0.004 | 0.006 | 0.038 | <0.001 | <0.001 |  |
| NTG (vs POAG) | 0.029 | 0.025 |  |  | 0.046 | 0.027 |  | 0.032 |  | 0.013 | 0.026 |  |  |  |  |
| **Time** |  |  |  |  |  |  |  |  |  |  |  |  |  |  |  |
| **Gender*time** |  |  |  |  |  |  |  |  |  |  |  |  |  |  |  |
| **MAP_0*time** |  |  |  | 0.0292 |  |  |  |  |  |  |  |  |  |  |  |
| **MAPdiff*time** |  |  |  |  |  |  |  |  |  |  |  |  |  |  |  |
| **Age*time** |  |  |  |  |  |  |  |  |  |  |  |  |  |  |  |
| **Diagnosis*time** |  |  |  |  |  |  |  |  |  |  |  |  |  |  |  |
| POAG (vs normal) |  |  |  |  |  |  |  |  |  |  |  |  |  | 0.001 |  |
| NTG (vs normal) |  |  |  | 0.015 |  |  |  | 0.031 |  |  |  |  |  |  |  |
| NTG (vs POAG) | 0.018 |  |  | 0.014 |  |  | 0.047 |  |  |  |  |  |  | 0.031 |  |

Only significant responses are reported. MAP_0: mean arterial pressure at baseline; POAG: Primary open angel glaucoma; NTG: Normal tension glaucoma; MAPdiff: difference of mean arterial pressure during examination; Peripapil: peripapillary mean; SUP: superior; Hemi: hemisphere. INF: inferior; TEM: temporal; NAS: nasal; NS NI IN IT TI TS ST SN as explained in the Materials and Methods section.

**Table S9. Macular total retinal thickness (OCT) adjusted linear mixed model estimates (µm).**

| **Region (ETDRS)** | **Center**  **(1)** | **TEM**  **(1-3)** | **SUP**  **(1-3)** | **NAS**  **(1-3)** | **INF**  **(1-3)** | **SUP Hemi**  **(1-3)** | **INF Hemi**  **(1-3)** | **All**  **(1-3)** | **TEM**  **(3-6)** | **SUP**  **(3-6)** | **NAS**  **(3-6)** | **INF**  **(3-6)** | **SUP Hemi**  **(3-6)** | **INF Hemi**  **(3-6)** | **All**  **(3-6)** | **SUP Hemi**  **(0-6)** | **INF Hemi**  **(0-6)** | **All**  **(0-6)** |
| --- | --- | --- | --- | --- | --- | --- | --- | --- | --- | --- | --- | --- | --- | --- | --- | --- | --- | --- |
| **Intercept** | 251.9 | 350.0 | 375.7 | 354.0 | 339.4 | 368.2 | 341.2 | 354.7 | 298.2 | 323.3 | 339.3 | 325.9 | 321.8 | 320.8 | 321.9 | 330.3 | 323.4 | 327.3 |
| **Gender** |  |  |  |  |  |  |  |  |  |  |  |  |  |  |  |  |  |  |
| **MAP_0** |  |  |  |  |  |  |  |  |  |  |  |  |  |  |  |  |  |  |
| **Age** |  |  |  |  |  |  |  |  |  |  |  |  |  |  |  |  |  |  |
| **Diagnosis** |  |  |  |  |  |  |  |  |  |  |  |  |  |  |  |  |  |  |
| POAG (vs normal) | | -24.0 | -19.8 | -18.1 | -27.6 | -19.5 | -25.2 | -22.4 | -21.8 | -23.4 | -34.9 | -29.6 | -23.5 | -28.3 | -27.5 | -21.8 | -25.5 | -25.3 |
| NTG (vs normal) | | -23.5 | -17.6 | -15.6 | -28.8 | -17.6 | -25.2 | -21.4 | -22.9 | -22.3 | -21.8 | -30.4 | -20.5 | -27.7 | -24.3 | -19.3 | -26.1 | -22.9 |
| NTG (vs POAG) |  |  |  |  |  |  |  |  |  |  |  |  |  |  |  |  |  |  |
| **time** | 19.6 |  |  | 31.2 |  |  |  | 31.0 |  |  |  |  |  |  |  |  |  |  |
| **Gender*time** |  |  |  |  |  |  |  |  |  |  |  |  |  |  |  |  |  |  |
| **MAP_0*time** |  | -0.3 | -0.3 | -0.3 | -0.3 | -0.3 | -0.3 | -0.3 |  |  |  |  |  | -0.1 | -0.1 | -0.1 | -0.1 | -0.1 |
| **MAPdiff*time** | -0.1 |  |  | -0.2 | -0.2 |  |  |  | 0.0 | 0.1 | -0.2 |  | 0.2 |  |  | 0.1 |  |  |
| **age*time** |  |  |  |  |  |  |  |  |  |  |  |  |  |  |  |  |  |  |
| **Diagnosis*time** |  |  |  |  |  |  |  |  |  |  |  |  |  |  |  |  |  |  |
| POAG (vs normal) | |  |  |  |  |  |  |  |  |  |  |  |  |  |  |  |  |  |
| NTG (vs normal) | |  |  |  |  |  |  |  |  |  |  |  |  |  |  |  |  |  |
| NTG (vs POAG) |  |  |  |  |  |  |  |  |  |  |  |  |  |  |  |  |  |  |

Regions are denoted by their radius in mm to the foveal center between parentheses. Only significant responses are reported.

MAP_0: mean arterial pressure at baseline; POAG: Primary open angel glaucoma; NTG: Normal tension glaucoma; MAPdiff: difference of mean arterial pressure during examination; TEM: temporal; SUP: superior; NAS: nasal; INF: inferior; Hemi: hemisphere.

**Table S10. Macular total retinal thickness (OCT) adjusted linear mixed model p-values.**

| **Region (ETDRS)** | **Center**  **(1)** | **TEM**  **(1-3)** | **SUP**  **(1-3)** | **NAS**  **(1-3)** | **INF**  **(1-3)** | **SUP Hemi**  **(1-3)** | **INF Hemi**  **(1-3)** | **All**  **(1-3)** | **TEM**  **(3-6)** | **SUP**  **(3-6)** | **NAS**  **(3-6)** | **INF**  **(3-6)** | **SUP Hemi**  **(3-6)** | **INF Hemi**  **(3-6)** | **All**  **(3-6)** | **SUP Hemi**  **(0-6)** | **INF Hemi**  **(0-6)** | **All**  **(0-6)** |
| --- | --- | --- | --- | --- | --- | --- | --- | --- | --- | --- | --- | --- | --- | --- | --- | --- | --- | --- |
| **Intercept** | <0.001 | <0.001 | <0.001 | <0.001 | <0.001 | <0.001 | <0.001 | <0.001 | <0.001 | <0.001 | <0.001 | <0.001 | <0.001 | <0.001 | <0.001 | <0.001 | <0.001 | <0.001 |
| **Gender** |  |  |  |  |  |  |  |  |  |  |  |  |  |  |  |  |  |  |
| **MAP_0** |  |  |  |  |  |  |  |  |  |  |  |  |  |  |  |  |  |  |
| **Age** |  |  |  |  |  |  |  |  |  |  |  |  |  |  |  |  |  |  |
| **Diagnosis** |  |  |  |  |  |  |  |  |  |  |  |  |  |  |  |  |  |  |
| POAG (vs normal) |  | <0.001 | 0.005 | 0.021 | <0.001 | 0.004 | 0.001 | 0.001 | <0.001 | <0.001 | <0.001 | <0.001 | <0.001 | <0.001 | <0.001 | <0.001 | <0.001 | <0.001 |
| NTG (vs normal) |  | <0.001 | 0.009 | 0.038 | <0.001 | 0.007 | 0.001 | 0.002 | <0.001 | <0.001 | 0.0156 | <0.001 | <0.001 | <0.001 | <0.001 | <0.001 | <0.001 | <0.001 |
| NTG (vs POAG) |  |  |  |  |  |  |  |  |  |  |  |  |  |  |  |  |  |  |
| **time** | 0.035 |  |  | 0.049 |  |  |  | 0.048 |  |  |  |  |  |  |  |  |  |  |
| **Gender*time** |  |  |  |  |  |  |  |  |  |  |  |  |  |  |  |  |  |  |
| **MAP_0*time** |  | 0.008 | 0.006 | 0.027 | 0.036 | 0.006 | 0.029 | 0.012 |  |  |  |  |  | 0.030 | 0.008 | 0.022 | 0.015 | 0.007 |
| **MAPdiff*time** | 0.040 |  |  | 0.018 | 0.046 |  |  |  | 0.027 | 0.005 | 0.011 |  | 0.001 |  |  | 0.024 |  |  |
| **age*time** |  |  |  |  |  |  |  |  |  |  |  |  |  |  |  |  |  |  |
| **Diagnosis*time** |  |  |  |  |  |  |  |  |  |  |  |  |  |  |  |  |  |  |
| POAG (vs normal) |  |  |  |  |  |  |  |  |  |  |  |  |  |  |  |  |  |  |
| NTG (vs normal) |  |  |  |  |  |  |  |  |  |  |  |  |  |  |  |  |  |  |
| NTG (vs POAG) |  |  |  |  |  |  |  |  |  |  |  |  |  |  |  |  |  |  |

Regions are denoted by their radius (mm) to the foveal center between parentheses. Only significant responses are reported.

MAP_0: mean arterial pressure at baseline; POAG: Primary open angel glaucoma; NTG: Normal tension glaucoma; MAPdiff: difference of mean arterial pressure during examination; TEM: temporal; SUP: superior; NAS: nasal; INF: inferior; Hemi: hemisphere.
